# Knockdown resistance (*kdr*) mutations in Indian *Anopheles culicifacies* populations

**DOI:** 10.1186/s13071-015-0946-7

**Published:** 2015-06-18

**Authors:** Cherry L. Dykes, Raja Babu S. Kushwah, Manoj K. Das, Shri N. Sharma, Rajendra M. Bhatt, Vijay Veer, Om P. Agrawal, Tridibes Adak, Om P. Singh

**Affiliations:** National Institute of Malaria Research, Sector 8, Dwarka, Delhi India; National Institute of Malaria Research Field Unit TB Sanatorium complex, Itki, Ranchi, India; National Institute of Malaria Research Field Unit, RLTRI campus, Raipur, India; Defence Research Laboratory, Tezpur, Assam India; School of Studies in Zoology, Jiwaji University, Gwalior, India

**Keywords:** *Anopheles culicifacies*, Voltage gated sodium channel, Knockdown resistance, Insecticide resistance

## Abstract

**Background:**

*Anopheles culicifacies s.l.* is one of the primary vectors of malaria in India responsible for the highest number of malaria cases. This vector is resistant to DDT in most parts of the country with indication of emerging resistance to pyrethroids. Since knockdown resistance (*kdr*) is known to confer cross-resistance between DDT and pyrethroids owing to a common target site of action, knowledge of prevalence of knockdown resistance (*kdr*) alleles is important from insecticide resistance management point of view.

**Methods:**

Nine populations of *An. culicifacies* belonging to five states of India, representing northern, western and central-east India, were screened for the presence of two alternative *kdr* mutations L1014F and L1014S using PCR-based assays. Dead and alive mosquitoes, following WHO standard insecticide susceptibility test against deltamethrin and DDT, were tested for allelic association.

**Results:**

L1014F mutation was recorded in all populations studied except from Haryana and Rajasthan states in northern India, with low frequencies ranging between 0.012 and 0.076; whereas presence of L1014S mutation was recorded in five populations only belonging to central-east India, with allelic frequencies ranging between 0.010 and 0.046. Both the *kdr* mutant alleles were found mostly in heterozygous condition without deviating from Hardy-Weinberg equilibrium. Both mutations showed protection against deltamethrin whereas only L1014S mutation showed protection against DDT when tested using additive model.

**Conclusions:**

The two L1014-*kdr* mutations, L1014F and L1014S, co-occurred in five populations belonging to Chhattisgarh and Odisha states of India whereas L1014F was present in all populations studied except populations from northern states. Both *kdr* mutations were found with very low allelic frequencies mostly in heterozygous condition and exhibited protection against deltamethrin.

## Background

*Anopheles culicifacies sensu lato* is one of the major malaria vectors in the Indian subcontinent accounting for 60–65 % malaria cases in India [1] mainly in rural and forested areas and prevalent in most parts of mainland India.

Vector control is an essential component of any malaria control programme. In India, control of malaria vectors, mainly in rural areas, relies on Indoor Residual Spray (IRS) and use of insecticide treated nets (ITN). Of the four insecticide groups approved for IRS, currently organochlorine (DDT), organophosphate (malathion) and synthetic pyrethroids (SP) are being used in India. Carbamates have yet not been introduced for public health sprays in India [2]. Resistance to DDT in *An. culicifacies* was reported as early as in the late sixties [3] and to malathion in 1973 [4] and currently this vector is resistant to DDT and malathion in most parts of India [5]. Synthetic pyrethroids are now being used to tackle DDT- and malathion-resistant mosquitoes either in the form of IRS or as impregnated mosquito nets. Pyrethroids are the only insecticide group, which have been recommended by World Health Organization (WHO) for treatment of mosquito nets due to their rapid knockdown effect and relatively lower mammalian toxicity [6]. Use of long-lasting insecticidal nets (LLIN), is now expanding in India, with the government’s target to cover most of the endemic areas having Annual Parasite Incidence (API) >5 [7]. Since alternative insecticides are not currently available to replace pyrethroids, judicious and conscientious use of pyrethroids is essential to prevent or slowdown the development of resistance.

DDT and pyrethroids are neurotoxins which act on the voltage-gated sodium channels (VGSC) by altering their gating kinetics, resulting in prolonged opening of individual channels leading to paralysis and eventual death of the insect [8]. Pyrethroids are still an effective insecticide group against *An. culicifacies* and reports of resistance against synthetic pyrethroids is scarce except in Surat district of Gujarat, which arose after the use of SP in the form of IRS and insecticide treated mosquito nets and possibly in the agricultural sector [9]. There is an urgent need of monitoring pyrethroid resistance across the country since the use of pyrethroids is expanding in public health as well as in the agricultural sector. The development of insecticide resistance will be a major setback to the national malaria control programme due to the unavailability of alternative insecticides, which are safe and cost effective. Pyrethroids are the best insecticides ever developed for public health use from the point of view of both safety and effectiveness. It is therefore essential to use this important group of insecticides judiciously and cautiously, with regular monitoring of the status of insecticide resistance in vector populations for an effective vector control programme.

Knockdown resistance (*kdr*) against DDT and pyrethroids is one of the resistance mechanisms in insects including anophelines. Knockdown resistance to DDT and the pyrethroids was first described in the housefly *Musca domestica* L. [10]. This trait confers reduced neuronal sensitivity to these insecticides and subsequently leads to development of cross-resistance to all synthetic pyrethroid insecticides [11]. The mutations that cause resistance are most commonly found in domain II region (between IIS4-5 linker and IIS6) of the VGSC protein where five residues have been implicated for resistance to date: Met918 in the IIS4-IIS5 linker, Leu925, Thr929 and Leu932 in IIS5 and Leu1014 in IIS6 [12, 13]. The L1014F/S in IIS6 which is referred to as *kdr* mutation, confers knockdown resistance phenotype in anophelines [14, 15] and has been reported in *Anopheles gambiae,* [16, 17], *Anopheles arabiensis* [18], *Anopheles stephensi* [19, 20], *Anopheles sinensis* and many other anophelines [21, 22]. Recently L1014F/S mutation was reported in *An. culicifacies* [23, 24]. However, the prevalence and incidence of these mutations in India is not known. We therefore surveyed various populations from India to study the distribution and frequency of *kdr* alleles in India.

## Methods

### Mosquito collection and processing

*Anopheles culicifacies* samples were collected from nine populations belonging to five states of India representing north (Haryana and Rajasthan states), west (Gujarat state) and central-east India (Chhattisgarh and Orissa states). These are: Sonepat (28.98° N and 77.02° E) district of Haryana, Alwar (27°26’N–27°29’N and 76°31’E–76°35’E) district of Rajasthan, Surat (21–22°N and 73–74°E) district of Gujarat, Malkangiri (17°45’N–18°40’N and 81°10’E–82°00’E) and Koraput (18° 10’N–20° 10’N, and 82° 10’E–83° 20’ E) districts of Odisha, Raipur (22°33’N–21°14’N and 82°6’E–81°38’E), Dantewada (18°46’N–19°28’N, and 80°15’E–81°58’E), Gidam (18.98°N and 81.40°E) and Bilaspur (21°47’N–23°8’N and 81°14’E–83°15’E) districts of Chhattisgarh, Adult female mosquitoes were collected from cattle sheds and human dwellings in the morning (6:00–8:00 AM) using a mouth aspirator and a flash torch. Mosquitoes from Dantewada and Malkangiri were transported to the laboratory and F_1_ progeny was obtained for bioassay with insecticides. A proportion of the mosquitoes from Koraput and Sonepat which had an appropriate gonotrophic stage (semi-gravid) for polytene chromosome squash preparation were processed for ovary extraction. Remaining mosquitoes were individually kept in a microcentrifuge tube containing a piece of silica gel.

For extraction of ovaries, blood-engorged field collected female mosquitoes were kept in a cage at room temperature for 6 to 10 h till attainment of semi-gravid (Christophers’ stage late III) condition. Ovaries from individual semi-gravid mosquitoes were extracted, preserved in modified Carnoy’s fixative (1:3 glacial acetic acid and methanol) and transported to the laboratory at Delhi. The remaining carcass of individual mosquitoes was preserved in isopropanol for DNA isolation.

### Species identification

#### Morpho-taxonomy

The adult mosquitoes collected from field were identified to species level (*sensu lato*) using keys by Christophers (1933) [25].

#### Sibling species identification

Squashed polytene preparations from ovarian nurse cells were prepared and stained following method described by Green and Hunt [26]. Polytene chromosome arrangement was checked for inversions present on chromosome X and 2 and identified at sibling species level using species-specific inversions which are ‘X+a+b, 2+g^1^+h^1^’ for Species A, ‘Xab, 2g^1^+h^1’^for Species B, ‘Xab, 2+g^1^h^1^’ for Species C and ‘X+a+b, 2i^1^+h^1^’ for Species D following Subbarao *et al.* [27]. Mosquitoes collected from Alwar were subjected to allele-specific PCR (ASPCR) for identification of sibling species by the method developed by Singh *et al.* [9, 28]. Species diagnostic multiplex PCR based on mitochondrial cytochrome oxidase subunit II (COII) [29] was not employed for sibling species identification in this study since the SNP markers earlier reckoned to be species specific were later found to be unreliable (Singh *et al.*, unpublished observation).

### Insecticide bioassay

Field collected *An. culicifacies* mosquitoes from Dantewada and Malkangiri were brought to the laboratory and F_1_ generation was obtained for insecticide bioassay. Bioassay was not carried out for other populations. Three-to-four day old and sugar-fed mosquitoes were exposed to insecticide impregnated papers using WHO’s standard insecticide susceptibility test kit. The insecticide impregnated papers used for bioassay were 4 % DDT and 0.05 % deltamethrin. Batches of 15–25 mosquitoes were transferred carefully into a holding tube lined with normal paper which was then transferred to an exposure tube lined with insecticide impregnated paper and finally transferred back to holding tube after 1-h exposure to the insecticide. Similarly, at least 20 mosquitoes were exposed to control papers in each experiment. The mosquitoes were provided with 10 % glucose soaked in a cotton pad and kept for 24 h in holding tube at room temperature for recovery. The dead and alive mosquitoes in each tube were separated and individually preserved in isopropanol for DNA isolation.

### DNA isolation

Prior to DNA isolation, except for those mosquitoes which were subjected to cytotaxonomy, one third of the abdomen of female mosquitoes containing spermatheca was removed to eliminate DNA content originating from sperms of sexual counterpart. DNA isolation from individual mosquito was carried out following the method by Livak [30].

### *kdr* genotyping

Genotyping of L1014-*kdr* mutations was done using Amplification Refractory Mutation System (ARMS) for L1014F mutation [23] and Primer Introduced Restriction Analysis PCR (PIRA-PCR) for L1014S mutation [24]. Other PCR-based assays (ASPCR and PIRA-PCR) developed by Singh *et al.* [23] for L1014F genotyping are no longer suitable after discovery of 1014S allele because one of the primers used in these assays will not anneal to 1014S allele resulting in null allele. Samples were not genotyped for V1010L mutation as this is linked to L1014S [23].

### DNA sequencing

A total of 15 samples from Dantewada were sequenced for domain II of the VGSC using primers KdrF and KdrR following Singh *et al.* [24] to validate *kdr-*genotyping result.

### Genetic analysis

Hardy-Weinberg equilibrium was tested based on exact tests with a Markov chain of 1,000,000 steps and 100,000 dememorization steps using software Arlequin ver 3.5 [31]. Allelic association studies were performed using Fisher’s exact test and odds ratio test.

## Results

The results of genotyping of mosquitoes for *kdr* alleles (wild, 1014 F and 1014S), distribution of different genotypes and allelic frequencies for 9 Indian populations are provided in Table 1. It was observed that *kdr* allele 1014 F was present in Surat, Malkangiri, Koraput, Bilaspur, Raipur and Dantewada with allele frequencies ranging between 0.012 and 0.074. The allele was absent from Alwar, and Sonepat. The highest allelic frequencies of 1014 F were noted in Dantewada and Malkangiri populations with allelic frequencies ranging between 0.071–0.074. The other allele 1014S was present in Malkangiri, Bilaspur and Raipur populations, but absent in Surat, Alwar and Koraput populations. The highest allelic frequencies of 1014S were found in Malkangiri and Dantewada, which are 0.045 and 0.046 respectively. In Malkangiri, Bilaspur, Gidam, Dantewada and Raipur both alleles (1014 F and 1014S) were present.

**Table 1:** Allelic frequencies of L1014, 1014 F and 1014S and in different populations of An. culicifacies in India

Locality	*n*	Genotypes						Allelic frequencies			HWE parameters		
		L/L	L/F	L/S	F/S	F/F	S/S	L1014	L1014F	L1014S	*H*_*O*_	*H*_*E*_	*p*
Surat (Gujarat)	186	167 (0.898)	18 (0.097)	0	0	1 (0.005)	0	0.946	0.054	0.000	0.0968	0.1020	0.4091
Malkangiri (Odisha)	90	71 (0.788)	11 (0.122)	8 (0.088)	0	0	0	0.894	0.061	0.044	0.1056	0.1013	0.9058
Koraput (Odisha)	76	71 (0.934)	5 (0.066)	0	0	0	0	0.967	0.033	0.000	0.0658	0.0640	1.0000
Bilaspur (Chhattisgarh)	100	91 (0.91)	7 (0.07)	2 (0.02)	0	0	0	0.955	0.035	0.010	0.0900	0.0871	1.0000
Raipur (Chhattisgarh)	43	41 (0.953)	1 (0.023)	1 (0.023)	0	0	0	0.977	0.012	0.012	0.0465	0.0462	1.0000
Dantewada PHC, (Chhattisgarh)	108	83 (0.769)	14 (0.130)	10 (0.093)	0	1 (0.009)	0	0.880	0.074	0.046	0.2222	0.2196	0.6353
Gidam PHC, (Chhattisgarh)	234	200 (0.855)	24 (0.102)	10 (0.043)	0	0	0	0.906	0.073	0.021	0.1453	0.1372	1.0000
Sonepat (Haryana)	15	15 (1.000)	0	0	0	0	0	1.000	0.000	0.000	0.0000	0.0000	1.0000
Alwar (Rajasthan)	82	81 (0.988)	0	0	0	0	0	0.994	0.000	0.000	0.0000	0.0000	1.0000

Figures in parenthesis indicate genotype frequencies

*n* number of samples assayed, *L* leucine, *F* phenylalanine, *S* serine, *HWE* Hardy-Weinberg equilibrium, *H*_*O*_ observed heterozygosity, *H*_*E*_ expected heterozygosity, *p* probability value

Sequencing results of representative samples of each genotype as determined by PCR-based assays (L/L = 5, L/S = 5, L/F = 4 and F/F = 1) were in agreement with PCR-based *kdr*-genotyping.

**Fig. 1:**
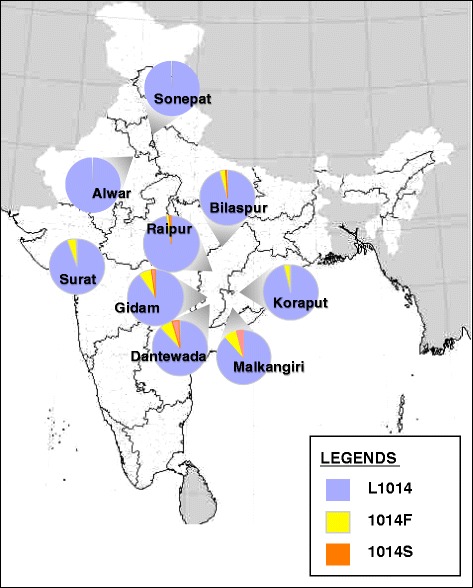
Frequency distribution of *kdr* alleles in *An. culicifacies* populations from different parts of India

Distribution of allelic frequencies of different L1014-alleles is presented in Fig. 1. The *kdr* mutant alleles in all populations were mostly in heterozygous condition with wild, without deviating from Hardy-Weinberg equilibrium.

A total of 44 samples of *An. culicifacies* were examined for polytene chromosome from two areas, 29 from Koraput, Odisha and 15 from Sonepat, Haryana (Table 2). Of the 29 samples examined for ovarian polytene chromosomes from Koraput, 20 were successfully identified as species B while all 15 samples from Sonepat were identified as species A. Among these, only one specimen of species B from Koraput was found with 1014-L/F genotype while the rest were L1014. All samples from Sonepat identified as species A were wild type for *kdr*. No ovaries could be collected from Alwar populations and therefore ASPCR assay which discriminates species A/D from species B/C/E was used to identify sibling species [28]. Of the 82 samples analyzed through ASPCR assay, 23 were identified as species B/C/E and 58 as species A/D. Only one specimen identified as species A/D was genotyped as L/S, the rest were wild (L/L).

**Table 2:** Distribution of L1014, 1014F and 1014S kdr alleles in different sibling species of An. culicifacies

A. Species identified by polytene chromosome examination																				
Locality	n	Species A						Species B						Species C						UI^a^
		L/L	L/F	L/S	F/S	F/F	S/S	L/L	L/F	L/S	F/S	F/F	S/S	L/L	L/F	L/S	F/S	F/F	S/S	
Koraput (Odisha)	29	-	-	-	-	-	-	19	1	-	-	-	-	-	-	-	-	-	-	9
Sonepat (Haryana)	15	15	-	-	-	-	-	-	-	-	-	-	-	-	-	-	-	-	-	
B. Species identified by ASPCR																				
Locality	n	Species A/D						Species B/C												
		L/L	L/F	L/S	F/S	F/F	S/S	L/L	L/F	L/S	F/S	F/F	S/S							
Alwar (Rajasthan)	82	58	0	1	0	0	0	23	0	0	0	0	0							

*n* number of samples assayed, *L* leucine, *F* phenylalanine, *S* serine

^a^ *UI* unidentified

The distribution of various *kdr* genotypes in dead and alive mosquitoes after 1-h exposure to deltamethrin (0.05 %) and DDT (4 %) has been shown in Table 3. Allelic association studies (additive model) using fisher’s exact test and odds ratio showed that L1014F and L1014S *kdr* mutations have significant protection against deltamethrin however, only L1014S showed significant protection against DDT. The results of statistical analyses have been shown in Table 3. The *kdr* factor is known to be recessive or incompletely recessive. However we failed to perform association studies using recessive model due to the absence of homozygotes for mutant *kdr* alleles.

**Table 3:** Association of kdr alleles with insecticide resistance

Locality	Insecticide used	Exposure time	*n*		Genotypes						Allelic association (additive model)			
											Fisher’s exact test		Odds ratio (95 % CI)	
					LL	LF	FF	LS	SS	FS	L vs. F	L vs. S	L vs. F	L vs. S
Dantewada (Chhattisgarh)	DEL (0.05 %)	1 h	294	Alive	30	13	0	7	0	0	*p* < 0.001	*p* < 0.01	5.03 (2.32–10.89)	4.66 (1.72–12.66)
				Dead	218	16	0	10	0	0				
Malkangiri (Odisha)	DDT (4 %)	1 h	90	Alive	29	6	0	7	0	0	NS	<0.05	1.67 (0.49–5.71)	9.15 (1.10–76.27)
				Dead	42	5	0	1	0	0				

*DEL* deltamethrin, *n* number of samples, *L* leucine, *F* phenylalanine, *S* serine, *CI* confidence interval, *p* probability value, *NS* non-significant

## Discussion

In this study, attempts were made to monitor the frequency distribution of *kdr* alleles in different populations of a major malaria vector *An. culicifacies* in India which revealed widespread presence of the two types of *kdr* mutations—1014 F and 1014S, the most common *kdr* mutations reported in insects including anophelines. However, regional differences were noticed both in terms of allelic frequencies as well as type of mutations present.

In a north Indian *An. culicifacies* population i.e. Alwar and Sonepat, none of the two *kdr* mutations were found. It is interesting to note that these populations comprised of species A or A/D. Species A has been reported to be comparatively susceptible to DDT as compared to species B [32] and has not been reported to be resistant to pyrethroids so far.

In Surat population (western India), only one mutation, i.e. 1014 F, was present with allelic frequency of 0.05. The Surat population was found to be resistant to DDT as well as pyrethroids in a study carried out by Singh *et al.* [9]. This is the population where a high level of pyrethroid resistance was reported for the first time with only 60–78 % mortalities with standard WHO susceptibility test and high knockdown time (KDT_50_ ranging from 74–81 min). The *An. culicifacies* population in this area comprised of species B and C only with preponderance of the former [9].

In central-eastern India, both *kdr* mutations (1014 F and 1014S) were observed in most of the populations such as Malkangiri of Odisha, Dantewada, Raipur and Bilaspur of Chhattisgarh except in Koraput of Odisha where only one mutation, i.e. 1014 F, was recorded. It was surprising to note that 1014S mutation was present in Malkangiri population and absent in Koraput both of which are geographically close with a distance of ~100 kms. However differences exist in sibling species composition in both areas where Koraput population comprised of species B only whereas Malkangiri population comprised of species B and C with preponderance of species B [24]. However, it will be misleading to conclude on the basis of this finding that 1014S is absent in species B because both species had both *kdr* mutations in Malkangiri [24].

So far we could not find *kdr* mutation in species A or D of *An. culicifacies* and both *kdr* mutations were noticed in populations with species B and C. However, it is premature to conclude that *kdr* mutations are found in species B and C only, due to limited data on species A and D.

The frequency of *kdr* mutations L1014F and L1014S was very low in the populations studied and these alleles were found mostly in heterozygous conditions with less than 1 % homozygotes. The alleles were, however, well in agreement with HWE (*p* = 0.4–1.0). This is contrary to a report by Hoti *et al.* [33] carried out in the same area, where the frequency of homozygous RR (1014 F) was too high (71 %) as compared to heterozygotes (4 %), resulting in significant departure from HWE (*p* = 0.00000, Fisher’s exact-test) due to severe deficiency of heterozygotes. One possible reason for this departure may be genotyping error due to several mismatches in flanking primers that were basically designed for *An. gambiae* and used for *An. culicifacies* due to non-availability of DNA sequence of VGSC for *An. culicifacies*.

Allelic association tests in this study showed that both L1014-*kdr* mutations had protection against deltamethrin but we did not observe protection by L1014F allele against DDT using additive model. The *kdr* factor is reportedly recessive [15] or incompletely recessive, [34] however, it was not possible to test the effect of *kdr* alleles on protection on insecticide resistance using recessive model due to lack of homozygous mutant alleles in our bioassay results. The exact nature of protection can best be studied in a population where sufficient numbers of homozygous individuals for *kdr* alleles are present. In this study area, the frequency of homozygous mutant alleles is extremely low (<1 %). Colonization of pure lines of *An. culicifacies* having different *kdr* mutations may be one alternative to establish phenotypic response of *kdr* alleles.

The present study reveals widespread presence of *kdr* alleles with very low frequencies. Thus *kdr* factor is not an important mechanism of resistance so far. However, in recent years there has been an increase in the use of ITNs in public health in India with an aim to cover the population under the risk of malaria with API >5. With increased use of pyrethroids in public health, such widespread presence of *kdr* alleles may result in their positive selection. In a longitudinal study made in Kenya, dramatic increase in *kdr* allele frequency in *An. gambiae* from 1996 through 2010 has been shown which coincided with the scale up use of insecticide-treated nets and by 2009–2010 the *kdr* L1014S allele was nearly fixed [35]. Regular monitoring on the relative role of *kdr-*based resistance and metabolic-detoxification mechanisms is important in a vector population where both DDT and pyrethroids are used for selection of appropriate insecticide to prevent or delay the occurrence of insecticide resistance.

## Conclusion

Two L1014-*kdr* mutations, L1014F and L1014S, co-occurred in five populations belonging to Chhattisgarh and Odisha states of India whereas L1014F was present in all populations studied except from Haryana and Rajasthan. Both *kdr* mutations were found with very low allelic frequencies mostly in heterozygous condition and exhibited significant protection against deltamethrin. Widespread presence of low frequencies of *kdr* mutations may lead to fixation of these alleles in presence of selection pressure. The data generated in this study will be helpful in the successful implementation of integrated vector management of *An. culicifacies*, a main malaria vector in India.
